# The role of self-determination theory and motivational interviewing in behavioral nutrition, physical activity, and health: an introduction to the IJBNPA special series

**DOI:** 10.1186/1479-5868-9-17

**Published:** 2012-03-02

**Authors:** Pedro J Teixeira, António L Palmeira, Maarten Vansteenkiste

**Affiliations:** 1 Interdisciplinary Centre for the Study of Human Performance, Faculty of Human Kinetics, Technical University of Lisbon, Lisbon, Portugal; 2 University Lusófona of Humanities and Technologies, Lisbon, Portugal; 3 Department of Psychology, Ghent University, Ghent, Belgium

## 

On the occasion of the 9^th ^Annual Meeting of the International Society of Behavioral Nutrition and Physical Activity (ISBNPA) in Lisbon (2009), a satellite meeting was organized in Sintra entitled *Self-Determination Theory and Motivational Interviewing in Behavioral Nutrition, Physical Activity, and Health*. The organizers of this small meeting (about 100 people attended) were interested in stimulating a focused discussion around the similarities, differences, and complementary of self-determination theory (SDT; [1]) and Motivational Interviewing (MI; [2]). This gathering was spurred by both a recent growth in applied health behavior research based in SDT [3], and by a continuing interest in exploring the mechanisms by which MI produces results in practice [4].

The links between SDT, a well-established theory of human motivation and behavior, and MI, a popular clinical method for evoking behavior change are multiple and have been explored before [5,6], leading many to think that a formal "marriage" - i.e., accepting SDT as "the theory of MI" and MI as the "intervention method of SDT" - would be just a matter of time. Both models are explicitly person-centered and process-oriented, both emphasize that optimal behavior change must involve deep personal commitment and engagement, and both stress that a positive emotional "climate", defined by genuine empathy and unconditional regard towards patients or clients is a necessary condition for the success of behavior change interventions, especially their long-term effects. Moreover, both SDT and MI appear to have at its center the concept of motivation, endorsing the development of "internal" motives and the need for patients to take responsibility for change, to the detriment of externally imposed goals, pressures, or a preponderance of reasons for change which are nor personally meaningful [7,8].

The interest in both MI and SDT has grown steadily over the past decades, with scholars and practitioners working in fields such as eating behavior (e.g., [9]), physical activity (e.g., [10]), and diabetes (e.g. [11]), becoming increasingly interested in exploring motivational dynamics. It is recognized that the motivation underlying patients' behavior change attempts provides them the necessary energy to actually undertake change and plays a key role in successful long-term outcomes. In fact, the issue of behavioral persistence is a critical one in the era of behavioral, preventive, and "lifestyle" medicine, with individuals increasingly called upon to manage or "self-regulate" their own health [12]. Short-lived change, such as what results from so many weight loss programs, is not what health practitioners and their patients are usually looking for.

According to SDT, although patients and clients might put some initial effort in change, lasting results are more likely to fail if it is not undergirded by the 'right' motives [13]. This implies that it is critical to move beyond merely considering a patient's level or intensity of motivation but also consider the quality of their motivation. In fact, motivation is conceived by SDT as a differentiated concept and a distinction is made between different types of motivation (autonomous relative to controlled), with some motivational subtypes being more desirable because they yield more positive outcomes than other types. Specifically, SDT researchers maintain that patients can best self-endorse change such that they willingly or volitionally pursue it rather than feeling seduced or pressured to make those changes. Nevertheless, and despite recent progress (e.g., [14]), SDT-based clinical interventions are still in their early stages of development and testing and it is fair to say that, at this time, the operational characteristics of an "SDT intervention" have not been fully elucidated. Progress in identifying and defining these characteristics will be useful and necessary vis-à-vis the goal of analyzing and classifying current MI interventions as to their "SDT-compatibility".

A clear emphasis on patient autonomy can also be observed within MI [15]. Based on their clinical experience, Miller and Rollnick have repeatedly stressed that clinicians can best maximize clients' sense of autonomy during counseling to promote long-term change [2]. Consequently, various counseling techniques developed within the context of MI have the direct aim of fostering patient autonomy [8]. The motivational practices of MI have high practical utility and, as a result, have been warmly welcomed and used by practitioners working in diverse fields (e.g., [16]). Yet, what has been lacking within MI is a strong theoretical foundation and a clearer description of its "active ingredients", that is, particular processes or mechanisms consistently involved in a MI intervention that help explain, and perhaps more importantly, help predict behavior change. In this respect, it is noticeable that MI leaders appear to have moved away from traditional psychological mechanisms such as intrinsic motivation or perceived autonomy as putative "mediators" of change (in MI interventions), focusing instead on more tangible or "non-specific" aspects such as the client's verbalization of "change talk" or therapist's skills affecting the relational tone between the counselor and the patient [4]. It might be worth exploring these processes at a deeper psychological and theoretical levels, including through the lens of SDT [17], to gain more accurate insight into the critical components and processes involved in a MI intervention.

Although ideally both frameworks would be merged with the aim of developing a well-grounded theory with high practical usefulness, presently SDT and MI still remain in two separate worlds. Many MI-inspired practitioners remain somewhat indifferent to theoretical constraints, and most SDT-based interventions have not explicitly used MI techniques (although some have). Similarly, SDT conferences and key SDT texts have only occasionally highlighted MI studies, while MI conferences have not featured SDT studies nor have MI scholars typically cited SDT in their research reports [18]. This special issue was conceived to discuss the virtues and challenges in bridging this gap. It brings together 6 original review articles written by scholars working in the field of SDT and/or MI. Two articles are more theory-focused [8,17], while each of the other four covered a separate domain, including health care [19], physical activity [14], weight management [18], and eating behavior [12], which are key application fields within the scope of ISBNPA. It is fair to say that most authors who contributed to this series are recognized more for their work in the field of SDT than in MI. Nevertheless and on balance, a good amount of attention was provided to both frameworks in virtually all papers. In addition, both the founding fathers of MI (i.e., William Miller and Stephen Rollnick) and SDT (i.e., Edward Deci and Richard Ryan) have written a commentary on this series of articles, providing their experienced perspectives as to where we stand and how this set of papers can impel the field forward.

We believe that this series clearly shows that there is potential for integration between SDT and MI. Yet, we hasten to say that a few steps are required before this potential is fully exploited. First, for MI and SDT to be merged, scholars working in both fields need to fully understand each others' concepts. Specifically, the search for complementarity and potential integration between MI and SDT may encourage scholars in both fields to define their core concepts (e.g., autonomy, motivation, ambivalence, resistance) in a more precise and clearer way. Both MI and SDT scholars have developed their own terminology and vocabularium over the past decades and it is critical to examine whether concepts that are referred to with different labels carry a similar meaning and if the same term used in both MI and SDT studies is interpreted differently. For instance, while autonomy is highly emphasized in both frameworks, a closer reading suggests that differences exist in how it is interpreted [7,17]. Such differences may yield important clinical implications and may give rise to the formulation of different hypotheses which require empirical testing. This special issue aims to contribute to this conceptual clarification, although substantial work still needs to be done.

A second important step to exploit the potential for integration between MI and SDT involves empirically testing hypotheses derived from both frameworks. To date, only in a couple of empirical studies (e.g., [20]) hypotheses derived from MI and SDT were simultaneously tested, the results of which are promising but represent only a starting point. Finally, we believe it is useful that SDT and MI researchers increasingly work together in common research projects, especially in applied and translational work. Designing, implementing, and evaluating interventions are critical juncture points where theory and practice should come together to bring about the best results.

As a final note to the readers of this Special Series, it should be kept in mind that the articles in this series were written largely independently from each other. Moreover, no editorial attempt was made to maximize internal consistency, avoid redundancy, or reconcile different viewpoints. Not surprisingly, an overall read of these 6 (plus 2) contributions confirms that there is in fact some degree of duplication in the description of a few key concepts, and that there are also slightly differing views among authors on some topics. However, instead of it being confusing or tedious, we find that having different experts address similar topics from their unique and independent perspectives will likely enrich readers' reflection on the subject of health behavior change, allowing for a more nuanced and a deeper level of understanding of a topic which is neither simple nor straight-forward.

## Competing interests

The authors declare that they have no competing interests.

## Authors' contributions

PJT, MV, and ALP co-wrote the manuscript. All authors read and approved the final manuscript.
